# Cerebellitis Associated with Influenza A(H1N1)pdm09, United States, 2013

**DOI:** 10.3201/eid2009.140160

**Published:** 2014-09

**Authors:** Maroun M. Sfeir, Catherine E. Najem

**Affiliations:** University of Miami Miller School of Medicine/Jackson Memorial Hospital, Miami, Florida, USA (M.F. Sfeir);; Roger Williams Medical Center, Providence, Rhode Island, USA (C.E. Najem)

**Keywords:** Influenza virus, viruses, central nervous system infection, influenza vaccine, A(H1N1)pdm09

**To the Editor:** Central nervous system (CNS) manifestations of influenza are uncommon, especially in adults (*1*,*2*), and influenza-associated cerebellitis is exceedingly rare: 8 cases have been reported (*3*–*7*; Technical Appendix). We describe a case of cerebellitis caused by influenza A(H1N1)pdm09 in an adult woman.

The 37-year-old female patient who sought medical care in Florida, United States, on January 5, 2013, described a 4-day history of intermittent fever of 38.5°C, generalized fatigue, diffuse headache, mild nonproductive cough, 3 episodes of vomiting, and decreased oral intake. On January 4, she experienced acute onset of ataxia and dysarthric speech with slurred pronunciation. She reported no contact with sick persons, recent travel, or exposure to pets or birds. She had a medical history of asthma since childhood, controlled by using montelukast tablets and inhaled steroids. The patient denied having ever received an influenza vaccination.

The patient appeared ill; her oral temperature was elevated at 38.3°C, but other vital signs were within normal limits (blood pressure 109/70 mm Hg; pulse rate 88 beats/minute; respiratory rate 15 breaths per minute; and oxygen saturation 98% at room air). Mucosal membranes appeared normal. No neck stiffness or palpable lymph nodes were noted. Results of heart examination were normal. Lungs were clear to auscultation, and the abdomen was soft, indicating no hepatosplenomegaly or palpable masses. No rash was seen. The neurologic examination revealed normal mental status but moderate ataxic dysarthria. Her cranial nerves were intact, and motor strength was 5/5 throughout. Results of a sensory examination were normal, and patient’s reflexes were largely intact; Babinski sign was absent. However, her coordination was bilaterally impaired in finger-to-nose testing, and her gait was notably broad-based and ataxic.

Laboratory test results showed a leukocyte count of 13.72 × 10^3^ cells/mm^3^; percentages of neutrophils and lymphocytes were within reference limits at 59% and 25%, respectively. Levels of electrolytes, liver enzymes, and creatine phosphokinase were within reference ranges. C-reactive protein level was below the limit of detection. A nonenhanced brain computed tomographic scan revealed no pertinent findings. Brain magnetic resonance imaging (MRI) revealed enlarged bilateral cerebellar hemispheres with evidence of hypointensity of the affected thoracic vertebral segment on T1 image and hyperintensity on the T2 image (Figure). A lumbar puncture drained clear and colorless cerebrospinal fluid (CSF) with an opening pressure of 15 cm of H_2_O. CSF analysis was pertinent, showing presence of erythrocytes (7.5/mm^3^) and elevated number of leukocytes (330/mm^3^ [13% neutrophils and 62% lymphocytes]). Glucose and protein levels in CSF were 61 mg/dL and 41 mg/dL, respectively. Blood and urine cultures were negative for pathogens. A chest radiograph did not show infiltrates. Bacterial culture, acid-fast smear, and culture of CSF were all negative. Blood and CSF tests for HIV syphilis, respectively, were nonreactive. However, reverse transcription PCR (RT-PCR) for influenza A(H1N1)pdm09 virus RNA was positive in the nasopharyngeal swab sample and CSF specimens, at a titer of 4.5 × 10^5^ and 671 RNA copies/mL, respectively. RT-PCR of CSF was negative for viruses, including herpes simplex, Ebstein-Barr, cytomegalovirus, West Nile, and herpes zoster.

**Figure:**
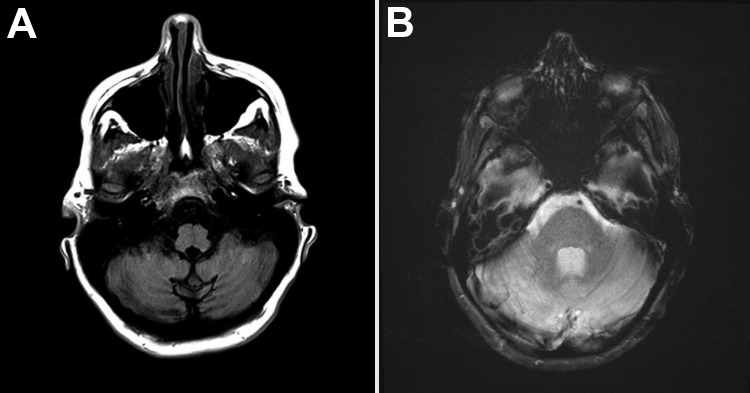
A) Magnetic resonance images of the brain of a woman with cerebellitis associated with influenza A(H1N1)pdm09, United States, 2013. T1-weighted axial MRI brain sequence showing hypo-intensity of bilateral cerebellar hemispheres. B) T2-weighted axial MRI brain sequence showing hyperintensity of bilateral cerebellar hemispheres.

The patient was given oseltamivir, 75 mg orally twice daily for 5 days. She experienced a progressive improvement of ataxia and dysarthria during her hospital stay and was discharged after 1 week. At a follow-up visit 2 months later, the patient had remained healthy and neurologically stable.

Cerebellitis, or acute cerebellar ataxia, is an inflammatory process of the cerebellar white matter that occasionally is manifested after systemic viral or bacterial infections (*8*). The following pathogens are known to cause acute cerebellitis: viruses varicella-zoster, herpes simplex, Epstein-Barr, rotavirus, echovirus, coxsackie, mumps, measles, and rubella; and bacteria *Borrelia burgdorferi*, *Coxiella burnetii*, *Salmonella typhi*, and *Bordetella pertussis* (*8*). Although the condition is presumed to be more common in children, adult cases of cerebellitis have been well described (*8*).

Before this case, influenza cerebellitis had been diagnosed in 8 cases as of 2011 (*3*–*7*) (Technical Appendix Table). Two cases were reported in adult women and the remaining were in children. Four had a probable diagnosis of influenza cerebellitis, although positive viral culture or RT-PCR was lacking (*4*). Seven case-patients had influenza-like illness preceding the neurologic symptoms (*3*–*6*). One case-patient showed evidence of pneumonia, and described the interval from respiratory illness onset to developing of cerebellar signs (*6*) Clinical sequelae, displayed in most case-patients affected by influenza cerebellitis (*3*,*4*,*6*,*7*), varied from complete recovery to development of serious complications such as hydrocephalus (*5*).

The pathogenic mechanism of influenza virus infection on the CNS can be either a direct invasion of the virus that causes acute illness or, more typically, a delayed autoimmune demyelinating postviral encephalopathy (*9*,*10*). Amplification of viral DNA in CSF is rare in most influenza-related CNS infections (*10*). In this case, the positive RT-PCR results for influenza A and the pertinent brain MRI findings, as well as the concurrent influenza prodromal symptoms, suggest that acute influenza cerebelllitis, rather than a postinfluenza encephalopathy, caused the associated neurologic findings.

The limitation of this report includes the lack of sequence data comparing the patient’s viral RNA from the CSF and the nasopharynx and the absence of sequential sampling during the course of her illness. In conclusion, influenza virus, though rare, should remain a consideration in patients who have acute cerebellitis during influenza season.

Technical Appendix Summary of published reported cases of influenza cerebellitis.
