# Supplementary material for: Cerebellitis Associated with Influenza A(H1N1)pdm09, United States, 2013
Source: Emerg Infect Dis. 2014 Sep;20(9):1578–80. doi: 10.3201/eid2009.140160 (PMC4178382; doi:10.3201/eid2009.140160)
Supplement: Technical Appendix — Summary of published reported cases of influenza cerebellitis. [file 14-0160-Techapp-s1.pdf]

DOI: http://dx.doi.org/10.3201/eid2009.140160

Cerebellitis Associated with Influenza A(H1N1)pdm09, United States, 2013

Technical Appendix Table. Characteristics of 8 reported cases of influenza cerebellitis in 5 published articles before this study. Six of the cases were described in children. Five patients had possible diagnosis of influenza cerebellitis. One patient had a complicated course.

Published case, year of publication, (Language of publication)	Characteristics, signs, symptoms	Brain imaging	CSF analysis	Confirmed (C) or probable (P) influenza cerebellitis	Lower respiratory tract symptoms and chest radiography findings	Treatment	Resolution of symptoms
Hayase Y et al, Internal Medicine 1997(English)	Woman, 31 y; fever and ataxia	Normal brain CT and MRI	Normal cell count, glucose and protein	(C) High serum hemagglutination inhibition titer to influenza B, and positive CSF RT-PCR for influenza B nucleoprotein gene	NA	NA	NA
Tlili-Graiess K et al, J Neuroradiol. 2006 (French)	4 children, 2-7 y; headache, fever, and vomiting; ataxia was present in 2 cases	Initial MRI (2 cases) demonstrated increased intensity on T2W and Flair sequences of the cerebellar gray matter	High lymphocytes and proteins in samples from 3 children; normal values for 1 child	(P) Viral serologic tests were negative for 3 cases; serum sample from 1 child was positive for Epstein-Barr virus	No respiratory symptoms noted. No chest radiograph	Prednisone ×5 d for 3 cases and ×10 d for 1 case.	Complete resolution of symptoms in 3 cases; persistent mild right upper limb paresis in 1
Apok V et al, J Neurol Neurosurg Psychiatry Poster 0102,2010 (English)	Teenaged girl with acute fulminant cerebellitis following a course of antiviral for H1N1 virus	Hydrocephalus	NA	(P) Influenza RT-PCR in CSF was negative	Patient had cough and fever and was started on antiviral therapy; no chest radiograph	NA	Residual left-sided ataxia after 3 months.
Hackett I et al, Ir Med J. 2013 (English)	Child, 6 y; headache, worsening dysarthria and ataxia; coordination revealed significant bilateral dysdiadochokinesis	MRI brain revealed findings consistent with a diagnosis of cerebellitis, no enhancement was noted post contrast	Lumbar puncture parameters were normal	(C) CSF samples RT-PCR–positive for influenza A and influenza B and nasopharyngeal aspirate RT-PCR–positive for influenza A(H1N1) and B.	2 weeks before treatment sought, patient had rhinitis, cough, and fever; chest radiography showed mild bilateral bronchial prominence	Oseltamavir ×5 d	All symptoms fully resolved after 1 week
Ishikawa T et al, Rinsho Shinkeigaku. 2006 (Japanese)	Woman, 25 y; fever and headache	T2-weighted brain MRI demonstrated a high signal lesion in the cerebellar cortex.123I-	Pleocytosis	(C) Nasal swab sample positive in the influenza assay and a ≥4× change in the antibody titer to influenza virus A	Not available	Oseltamivir	Truncal ataxia normalized after 3 mo.†

Published case, year of publication, (Language of publication)	Characteristics, signs, symptoms	Brain imaging	CSF analysis	Confirmed (C) or probable (P) influenza cerebellitis	Lower respiratory tract symptoms and chest radiography findings	Treatment	Resolution of symptoms
		IMP-SPECT showed hypoperfusion in the cerebellum		(H3N2) detected by hemagglutination inhibition			

*NA: not available.
†Followup imaging showed cerebellar cortical lesion observed on MRI had resolved 80 days after hospitalization; laboratory data indicated that cerebrospinal fluid pleocytosis had normalized ≈3 months later.
